# Revising the paradigm: Are bats really pathogen reservoirs or do they possess an efficient immune system?

**DOI:** 10.1016/j.isci.2022.104782

**Published:** 2022-07-19

**Authors:** Maya Weinberg, Yossi Yovel

**Affiliations:** 1 School of Zoology, Faculty of Life Sciences, Tel Aviv University, Tel Aviv 6997801, Israel; 2 Sagol School of Neuroscience, Tel Aviv University, Tel Aviv 6997801, Israel

**Keywords:** Immunology, Virology

## Abstract

While bats are often referred to as reservoirs of viral pathogens, a meta-analysis of the literature reveals many cases in which there is not enough evidence to claim so. In many cases, bats are able to confront viruses, recover, and remain immune by developing a potent titer of antibodies, often without becoming a reservoir. In other cases, bats might have carried an ancestral virus that at some time point might have mutated into a human pathogen. Moreover, bats exhibit a balanced immune response against viruses that have evolved over millions of years. Using genomic tools, it is now possible to obtain a deeper understanding of that unique immune system and its variability across the order Chiroptera. We conclude, that with the exception of a few viruses, bats pose little zoonotic danger to humans and that they operate a highly efficient anti-inflammatory response that we should strive to understand.

## Introduction

Bats (Chiroptera) comprise the only order of mammals with the ability for powered flight, and with nearly 60 million years of physiological adaptions for this ability (Lei and Dong, 2016). With over 1,400 species, bats account for more than 20% of all mammalian species, second only to rodents, and can be found everywhere on earth except the poles (Calisher et al., 2006). Bats play an important role in insect control, reseeding deforested areas, and pollinating a variety of plants (Boyles et al., 2011; Zaho, 2020). Despite these useful roles, bats are mostly perceived as posing a threat to public health, as major transmitters of pathogenic and potentially zoonotic viruses (Dobson, 2005; Leroy et al., 2005; Li et al., 2005; Calisher et al., 2006). COVID-19 is only one recent example of media reports (Zhou et al., 2020) connecting bats to human disease and targeting them as reservoir animals, despite a lack of evidence (Andersen et al., 2020). Although the coronavirus isolated from bats in Wuhan (China) was found to be 96% genetically identical to the beta coronavirus that started the current pandemic, this degree of similarity accounts for a temporal distance of several to many years between the two, when taking the mutation rate of the virus into account (Boni et al., 2020; Ruiz-Aravena et al., 2022). Notably, the receptor-binding domain (RBD) of the bat virus cannot bind to human cells, indicating that it is not the direct source of the pandemic (Andersen et al., 2020; Chan et al., 2020; Ruiz-Aravena et al., 2022). Although there is some evidence that the potential ancestral COVID-19 virus had originated in bats (Shereen et al., 2020), to date, two years after the pandemic first struck, we still do not know the direct source of the human pathogenic COVID-19 variant (Ruiz-Aravena et al., 2022; Frutos et al., 2022). The bats’ widespread image as a danger to public health will, however, be difficult to rehabilitate (Zaho, 2020; MacFarlane and Rocha, 2020). In this review, we scrutinized the literature in order to assess the evidence and determine whether bats are or are not reservoir animals for more than a hundred pathogenic viruses, as is often claimed (Calisher et al., 2006; Epstein and Newman, 2011; Hayman, 2016; Wang and Anderson, 2019). Our findings suggest that in many cases the confidence regarding the bats’ role as reservoir animals is not sufficiently supported. Although we do not claim that bats are never the origin of human pathogens, we suggest that their role has been consistently exaggerated and often without the necessary scientific basis.

## Are bats viral reservoir animals?

A reservoir animal is defined as an epidemiologically **connected population** in which the **pathogen** can be **permanently maintained** and from which **infection is transmitted** to the target population (Haydon et al., 2002). A slightly broader interpretation of this term is discussed by Ashford (Ashford, 2003).

More than 4,100 bat-associated viruses from 23 viral families were detected in ∼200 bat species (Chen et al., 2014). Of these viruses, more than 100 were identified as important for “emerging and re-emerging human infections” (Calisher et al., 2006; Wong et al., 2007). As we will show later in discussion, however, in a substantial proportion of these cases there is no sufficient evidence to consider bats the reservoir species of these viruses.

The minimum requirement for determining a reservoir species is the isolation of the relevant pathogen from the species’ population. However, a broad literature review revealed that in ∼50% of the reported human pathogenic viruses, an *identical* and *viable* pathogen was never isolated from bats. Ebola presents such an example, as bats are often accused of being reservoirs of this disease. The first study declaring fruit bats as reservoir animals of Ebola (Leroy et al., 2005) found that in bats in which immunoglobulin-g (IgG) specific for Ebola was detected, the only PCR-positive organs were the liver and spleen; levels of viral RNA were low, and no live virus was isolated. In other blood-filled organs (heart, liver, kidneys), no viral RNA was detected at all. This raises questions about the ability of the virus to be shed in bat bodily fluids. On the other hand, bats that were found to be positive for viral detection, using PCR tests, showed no IgG specific for Ebola in their serum. Thus, bats were found to be either viral carriers and sick or healed and immune (IgG positive with no virus detected). The authors themselves refer to this duality as “surprising.” In theory, the bats might have been tested shortly post-infection, at the stage in which the virus had been eradicated by the immune system, and the IgG titer had already increased. However, it is unlikely that all the bats were surveyed exactly at this time point. The more parsimonious explanation is that the bats were either sick or had overcome the disease and were now immune. We note, moreover, that PCR testing might not in itself be sufficient to detect an actual identical human pathogen virus. None of the later studies found that bats permanently host a viable Ebola virus. Moreover, the seroprevalence of Ebola antibodies in bat population is quite low—∼3% (Yuan et al., 2012; Olival et al., 2013). Many epidemiologists would argue that such a low prevalence is insufficient for considering the species a reservoir animal (Scott, 2001; Drexler et al., 2014; Markotter et al., 2020; De Oliveira and Bonvicino, 2020). In comparison, if we take a known case of a reservoir animal, such as birds and avian flu or the West-Nile virus, we expect to find much higher rates of seroprevalence as well as to find both IgG and the isolation of viable viruses (Shortridge et al., 1998; Alexander, 2007; Travis, 2008; Wodak et al., 2011). To date, however, although the source of the Ebola virus remains unknown (Kock et al., 2019), bats are routinely accused of being reservoir animals of this disease in numerous scientific publications (Wang, 2009; Schountz, 2014; Han et al., 2015; Woo and Lau, 2019; Banerjee et al., 2020). The deep molecular, immunological, and ecological gaps in the Ebola reservoir hypothesis are well summarized by (Leendertz et al., 2016). Those authors also point out several important sampling biases as well as a lack of scientific publications of essential negative results (i.e., cases where no evidence for carrying Ebola was found). A similar pattern characterizes the perceived connection between bats and severe acute respiratory syndrome (SARS) (Li et al., 2005; Wang et al., 2006; Ge et al., 2013). Although the coronavirus diversity seems to be higher in bats than in any other mammals, and viruses closely related to SARS-CoV, MERS-CoV, and HCoV-229E exist in bats, identical human pathogens have never been found in bats. In the case of SARS, a virus 95% similar to the human pathogen and which can infect the human cell line was isolated from a bat, but the actual human pathogen SARS was never isolated, despite intensive attempts (Chinese SARS Molecular Epidemiology Consortium, 2004; Poon et al., 2004; Hui and Zumla, 2019). Moreover, it is widely accepted that even if the transmission of SARS to humans originated in bats, it was indirect, and first transmitted to an intermediate host (civet cats). Accordingly, Drexler et al. (Drexler et al., 2014) state that the “Lack of bat coronavirus isolates and full genomes challenge taxonomic classification and mechanisms of putative host switches from bats into humans are unknown.” Thus, although the wildlife origin of SARS remains unknown to date (Wang et al., 2006; Andersen et al., 2020), bats are routinely blamed for spreading this virus to humans.

Although the definition of a reservoir animal refers to carrying the actual pathogen and not to a related virus, we accept the rationale that a closely related virus that is only a few mutations away from the target pathogen could make its carrier a reservoir. In most cases, however, there is no good evidence that this is, indeed, the situation.

To examine the general situation, we performed a meta-analysis of the literature and examined the finding for over 100 viruses for which bats have been considered potential reservoirs (Calisher et al., 2006; Wang and Cowled, 2015; Hayman, 2016). We found that in a significant proportion of the cases (48%) this claim has been based on the seroprevalence of antibodies or PCR tests, and not on actual virus isolation (Table 1). Moreover, many of the reported isolations are unconvincing: (1) Several viruses were only isolated from a single individual bat (Charlier et al., 2002); (2) In some cases isolation was performed from a homogenate of internal tissues from which transmission is unlikely (e.g., the liver and spleen) and not from oral swabs or saliva glands, urine, feces, or even blood or sera. (Mortlock et al., 2015; Hayman, 2016); (3) Several of the local viruses were also isolated from other animals in the region, including non-bat-specific ectoparasites (Ramírez-Martínez et al., 2021); and (4) Some isolations were taken from sick or dead individuals (Osborne et al., 2003; Kuzmin et al., 2010), which would probably not have transmitted the disease—sick bats have been shown to remain in the roost and refrain from social interactions (Moreno et al., 2021). Seroprevalence by itself doesn’t reflect the ability or even the potential for being a reservoir or creating spillover events (Barrantes Murillo et al., 2022).

**Table 1:** A literature analysis of 101 viruses for which bats were claimed to be reservoir hosts

No	Virus	Family, genus	Serology	PCR	Isolation	Interesting statements from the original paper	Ref
1	Kolente virus	Alpharhabdovirinae, Ledantevirus	no	no	yes		(Ghedin et al., 2013)
2	Tacaribe viru	Arenaviridae, Bunyavirales	yes	no	yes	It has not been found to infect humans	(Price, 1978b; Downs et al., 1963)
3	Nepuyo virus	Bunyaviridae, Bunyavirus	no	no	no	Most closely related to Nepuyo virus	(Calisher et al., 1971)
4	Guama virus	Bunyaviridae, Bunyavirus	no	no	no		(Epstein and Newman, 2011)
5	Catu virus	Bunyaviridae, Bunyavirus	no	no	no		(Pierlé et al., 2015)
6	Hanta virus	Bunyaviridae, Hantavirus	yes	yes	no	paper 1: Serotype of the isolates is closely related to Hantaan virus \ paper 2: sixteen samples positive were encountered among the wild rodents, bats, and opossums	(Kim et al., 1994)
7	Toscana virus	Bunyaviridae, Phlebovirus	no	no	yes	flies—rather than vertebrates—are considered to be its natural reservoir host. Isolated once from bat brain	(Charrel et al., 2005)
8	Rift valley fever virus	Bunyaviridae, Phlebovirus	yes	no	yes		(Kading et al., 2018; Boiro, Konstaninov and Numerov, 1987)
9	Kaeng Khoi virus	Bunyaviridae, unassigned	yes	no	yes	Taken from premature or dead bats. Found in bedbugs as well.	(Osborne et al., 2003; Williams et al., 1976)
10	Japanaut virus	Bunyaviridae, unassigned	no	yes	no		(Fagre et al., 2019; Ramírez-Martínez et al., 2021)
11	Ife virus	Bunyaviridae, unassigned	yes	no	yes		(Kemp et al., 1988; Fagre et al., 2019)
12	Fomede virus	Bunyaviridae, unassigned	yes	yes	no		(Fagre et al., 2019)
13	Bangui virus	Bunyaviridae, unassigned	no	no	yes		(Mourya et al., 2014)
14	SARS virus	Coronaviridae, Betacoronavirus	no	no	no	The overall nucleotide sequence identity between Sars Like-CoV and SARS-CoV T was 94%.	(Drexler et al., 2014; Banerjee et al., 2019)
						we suggest caution with conclusions on the zoonotic potential of bat viruses, based only on genomic sequence data. Viruses closely related to SARS-CoV, MERS-CoV and HCoV-229E exist in bats. Mechanisms of putative host switches from bats into humans are unknown.	
15	SARS like Corona virus	Coronaviridae, Betacoronavirus	yes	yes	no	The overall nucleotide sequence identity between SL-CoV and SARS-CoV T was 94%	(Li et al., 2005; Lau et al., 2005)
16	MERS virus	Coronaviridae, Betacoronavirus	no	yes	no		(De Wit et al., 2016; Anthony et al., 2017)
17	MERS like Corona virus	Coronaviridae, Betacoronavirus	no	yes	yes		(Ithete et al., 2013; Lau et al., 2021)
18	Other Corona virus in bats	Coronaviridea,	no	yes	?	We identify critical gaps in knowledge of bat coronaviruses, which relate to spillover and pandemic risk, including the pathways to zoonotic spillover	(Ruiz-Aravena et al., 2022)
19	Reston Ebola virus	Filoviridae, Ebolavirus	yes	yes	no		(Jayme et al., 2015)
20	Zaire Ebola virus	Filoviridae, Ebolavirus	yes	yes	no		(Leroy et al., 2005; Leendertz et al., 2016)
21	Bombali virus	Filoviridae, Ebolavirus	no	yes	no		(Forbes et al., 2019)
22	West Nile virus	Filoviridae, Flavivirus	yes	no	yes	Considered accidental infection.	(Paul, Rajagopalan and Sreenivasan, 1970)
23	Marburg	Filoviridae, Marburgvirus	yes	yes	yes	It can be concluded that there was no evidence of vertical transmission of infection in R. aegyptiacus. Virus isolated from spleen and liver however, virus was not detected in feces or urine collected from infected specimens or the cave floor	(Towner et al., 2009; Leendertz et al., 2016)
24	Lloviu virus	Filoviridea, Cuevavirus	yes	yes	yes		(Kemenesi et al., 2022)
25	Yokose8 virus	Flaviviridae, Flavivirus	yes	no	yes	Not associated with disease in either bats or humans	(MacKenzie and Williams, 2009; MacKenzie and Williams, 2009; Tajima et al., 2005)
26	Yellow fever virus	Flaviviridae, Flavivirus	yes	no	no		(Price, 1978b)
27	Uganda S virus	Flaviviridae, Flavivirus	no	no	yes		(Lumsden, Williams and Mason, 1961)
28	Tamana bat virus	Flaviviridae, Flavivirus	yes	no	yes		(Price, 1978a)
28	St Louis encephalitis virus	Flaviviridae, Flavivirus	yes	no	no	Were shown to be susceptible to infection	(Sulkin et al., 1963; Boiro, Konstaninov and Numerov, 1987)
29	Sokuluk virus	Flaviviridae, Flavivirus	no	no	yes	Pathogenic for suckling mice and mice weighing 8g only when inoculated intracerebrally.	(Sehreber et al., 1973)
30	Saboya virus	Flaviviridae, Flavivirus	no	no	no		(Varelas Wesley and Calisher, 1982)
31	Rio Bravo virus	Flaviviridae, Flavivirus	yes	no	yes	Not associated with disease in either bats or humans	(MacKenzie and Williams, 2009; Price, 1978a)
32	Phnom-Penh bat virus	Flaviviridae, Flavivirus	no	no	yes	Not associated with disease in either bats or humans	(MacKenzie and Williams, 2009)
33	Montana Myotis leukoen-cephalitis (MML) virus	Flaviviridae, Flavivirus	no	no	yes	A single isolation in 1960	(Charlier et al., 2002)
34	Kyasanur Forest disease virus	Flaviviridae, Flavivirus	yes	no	no		(Ajesh, Nagaraja and Sreejith, 2017; Pavri and Singh, 1968)
35	Kampar virus	Flaviviridae, Flavivirus	no	no	no	Although the exact origin of Kampar virus is unknown, epidemiological tracing revealed that the house of the index case is surrounded by fruit trees frequently visited by fruit bats. There is a high probability that Kampar virus originated from bats and was transmitted to humans via bat droppings or contaminated fruits.	(Chua et al., 2008)
36	Jugra virus	Flaviviridae, Flavivirus	no	no	yes		(MacKenzie and Williams, 2009)
37	Japanese encephalitis virus	Flaviviridae, Flavivirus	no	no	yes		(Sulkin et al., 1970; Cross et al., 1971)
38	Ileus virus	Flaviviridae, Flavivirus	yes	no	no	The main reservoir hosts are birds	(Stone et al., 2018)
39	Entebbe bat virus	Flaviviridae, Flavivirus	no	no	yes	Not associated with disease in either bats or humans	(Simpson et al., 1968; MacKenzie and Williams, 2009)
40	Dakar bat virus	Flaviviridae, Flavivirus	no	no	yes		(Simpson et al., 1968)
41	Central European encephalitis virus	Flaviviridae, Flavivirus	yes	no	no	unidentified bat	(Calisher et al., 2006; Kozuch et al., 1990; Kuno, 2001)
42	Carey Island virus	Flaviviridae, Flavivirus	no	no	yes	Not associated with disease in either bats or humans	
43	Bukalasa bat virus	Flaviviridae, Flavivirus	no	no	yes		(MacKenzie and Williams, 2009)
44	Dengue virus	Flaviviridae, Flavivirus	yes	yes	no		(Simpson et al., 1968)
45	Zika Virus	Flaviviridae, Flavivirus	no	yes	no		(Torres-Castro et al., 2021)
46	Hepacivirus	Flaviviridea, Hepacivirus	no	yes	no		(Platt et al., 2000)(Zhang, Yang and Li, 1998)
47	Pegivirus	Flaviviridea, Pegivirus	no	yes	no	Previously isolated in other mammals	(Quan et al., 2013)
48	Parixa virus	Herpesviridae, unassigned	no	yes	no		(Quan et al., 2013)
49	Herpes virus (gamma and beta)	Herpesviridae, unassigned	no	yes	no		(Calisher et al., 2006; Razafindratsimandresy et al., 2009)
50	Agua Preta virus	Herpesviridae, unassigned	no	yes	no		(Wibbelt et al., 2007; Razafindratsimandresy et al., 2009)
51	A cytomegalovirus	Herpesviridae, unassigned	no	no	no	Based on histology and microscopy	(Carnieli et al., 2016)
52	Influenza virus A	Orthomyxoviridae, Influenzavirus A	yes	no	no		(Tandler, 1996)
53	Nipah virus	Orthoparamyxovirinae, Henipavirus	yes	no	yes		(Lvov et al., 1979; Kelkar et al., 1981)
54	Hendra virus	Orthoparamyxovirinae, Henipavirus	yes	no	yes	Humans acquired the infection solely from horses to date and not from flyingfoxes. The route of transmission to humans probably occurs through contact with respiratory secretions of infected horses.	(Yob et al., 2001; Chua et al., 2002)
55	Parainfluenza virus type 2	Orthoparamyxovirinae, Respirovirus	yes	no	yes		(Halpin et al., 2000; Field et al., 2001)
56	Parainfluenza virus type 4	Orthoparamyxovirinae, Respirovirus	no	yes	no		(Pavri et al., 1971)
56	Sosuga virus	Paramyxoviridae, Pararubulavirus	no	yes	no		(Hause et al., 2021)
57	Tioman virus	Paramyxoviridae, Rubulavirus	no	no	yes	One paper found virus other paper found antibodies. Human infections due to Tiomanvirus have not been described	(Amman et al., 2015)
58	Menagle virus	Paramyxoviridae, Rubulavirus	yes	no	no		(Chua et al., 2001)
							(Breed et al., 2010)
59	Mapuera virus	Paramyxoviridae, Rubulavirus	no	no	yes		(Breed et al., 2010; Philbey et al., 1998)
60	Yogue virus	Bunyaviridae, Nairovirus	no	no	yes		(Henderson et al., 1995)
61	Mojui dos Campos virus	Picornaviridae, unassigned	no	no	yes		(Walker et al., 2015)
62	Kasokero virus	Bunyaviridae, Nairovirus	no	no	yes		(Wanzeller et al., 2002)
64	Issyk-kul	Bunyaviridae, Nairovirus	no	no	yes		(Kalunda et al., 1986; Walker et al., 2015)
65	Keterah virus	Bunyaviridae, Nairovirus	no	no	no	Isolation from bat blood of parasiting tick	(Walker et al., 2015; Lvov et al., 1973)
66	Gossas virus	Bunyaviridae, Nairovirus	no	no	yes		(Varma and Converse, 1976)
67	Picorna viruses (11 subspeicies)	Picornaviridae, varied	no	yes	no	Fecal samples of bats	(Walker et al., 2015)
68	Juruaca virus	Picornaviridae, unassigned	no	no	no	Unidentified bat	(Kemenesi et al., 2015; Yinda et al., 2017)
69	Bukakata virus	Reoviridae, Orbivirus	no	no	yes		(Epstein and Newman, 2011)
70	Pulau virus	Reoviridae, Orthoreovirus	no	no	yes	The transmissibility and pathogenicityof these viruses to humans are unknown	(Fagre et al., 2019)
71	Nelson Bay virus	Reoviridae, Orthoreovirus	no	no	yes	The transmissibility and pathogenicityof these viruses to humans are unknown	(Pritchard et al., 2006)
72	Melaka virus	Reoviridae, Orthoreovirus	no	no	no		(Gard and Compans, 1970)
73	Broome virus	Reoviridae, Orthoreovirus	no	yes	no		(Kaw et al., 2007)
74	Xi River virus	Reoviridae, Orthoreovirus	no	no	yes		(Thalmann et al., 2010)(Van Vuren et al., 2016)
75	Mahlapitsi virus	Reoviridae, Orthoreovirus	no	yes	no		(Harima et al., 2020)
75	Fikirini virus	Rhabdoviridae, Ledantevirus	no	no	yes		(Du et al., 2010)
76	West Caucasian bat viruses	Rhabdoviridae, Lyssavirus	no	yes	no	Have not been shown to cause human infections to date	(Kading et al., 2013)
77	Taiwan bat lyssavirus	Rhabdoviridae, Lyssavirus	no	yes	yes	Isolated from dead bats	(Coertse et al., 2020)
78	Shimoni bat lyssavirus	Rhabdoviridae, Lyssavirus	no	no	yes	Isolated from a dead bat	(Hu et al., 2018)
79	Rabies8 virus, (genotype 1)	Rhabdoviridae, Lyssavirus	no	no	yes	Most human rabies deaths are due to exposure to rabid dogs and rabies virus infection. The predominant reservoir of all the other lyssaviruses (rabies-related lyssaviruses) is Old World bats with RABV only associated with bats in the New World (Markotter and Coertse, 2018).	(Kuzmin et al., 2010)
80	Mokola virus	Rhabdoviridae, Lyssavirus	yes	no	no		(Markotter and Coertse, 2018)
81	Lagosbat virus	Rhabdoviridae, Lyssavirus	yes	yes	yes	Over 5000 individual bats have been tested in Africa with a detection rate of less than 1%. Have not been shown to cause human infections to date	(Markotter and Coertse, 2018; Wright et al., 2010)
82	Khujand virus	Rhabdoviridae, Lyssavirus	yes	no	yes	Have not been shown to cause human infections to date	
							(Boulger and Porterfield, 1958; Freuling et al., 2015; Coertse et al., 2021)
83	Irkut virus	Rhabdoviridae, Lyssavirus	yes	no	yes	Have not been shown to cause human infec-tions to date. Single bat isolation from brain	(Kuzmin et al., 2006; Kuzmin et al., 2003)
84	European batlyssaviruses 2	Rhabdoviridae, Lyssavirus	yes	yes	yes		(Kuzmin et al., 2003; Liu et al., 2013)
85	European batlyssaviruses 1	Rhabdoviridae, Lyssavirus	yes	yes	no		(Whitby et al., 2000; Brookes et al., 2005; Mcelhinney et al., 2013)
86	Duvenhage virus	Rhabdoviridae, Lyssavirus	yes	yes	yes	Each detection (antibodies, PCR, viral isolation) comes from a different study as quoted	(Serra-Cobo et al., 2002; Van Der Poel et al., 2005; Mcelhinney et al., 2013)
87	Australian bat lyssavirus	Rhabdoviridae, Lyssavirus	yes	no	yes		(Coertse et al., 2020); Markotter et al., 2013; Foggin, 1988)
88	Arvan virus	Rhabdoviridae, Lyssavirus	yes	no	yes	Have not been shown to cause human infections to date	(Prada et al., 2019; Fraser et al., 1996)
89	Oita 296 virus	Rhabdoviridae, unassigned	no	no	yes		(Kuzmin et al., 2006; Kuzmin et al., 1992)
90	Mount Elgon bat virus	Rhabdoviridae, unassigned	no	no	yes	It has not been associated with human infections	(Ghedin et al., 2013)
91	Kern canyon virus	Rhabdoviridae, unassigned	no	no	yes		(Murphy et al., 1970)
92	Vesicular stomatitis virus	Rhabdoviridae, Vesiculovirus	no	no	no	Experimental infection	(Blasdell et al., 2015)
93	Chandipura virus	Rhabdoviridae, Vesiculovirus	no	no	no	Sandflies were believed to be the vector of Chandipura virus but it has not been found in association with bats	(Donaldson, 1970)
94	Rubulavirus	Rubulavirinae, Orthorubulavirus	no	no	yes		(Donaldson, 1970)
95	Ross river virus	Togaviridae, Alphavirus	no	no	yes	Isolation from pool of 37 bats brains in China. In Australia where it is active bats are not considered as reservoir and no Isolation found.	(Baker et al., 2013a, 2013b)
96	Western equine encephalitis virus (WEEV)	Togaviridae, Alphavirus	no	no	no		(Zhao et al., 1997; Kay and Aaskov, 2020; Lau et al., 2017)
97	Venezuelan equine encephalitis virus(VEEV)	Togaviridae, Alphavirus	no	yes	yes	Bat and rodent species were PCR positive	(Sotomayor-Bonilla et al., 2017)(Correa-Giron, Calisher and Baer, 1972)
98	Sindbis virus	Togaviridae, Alphavirus	no	no	yes	Seems to circulate mostly in birds	(Blackburn et al., 1982)
99	Chikungunya virus	Togaviridae, Alphavirus	no	no	yes	Together with NHPs, some bats and rodents, as well as ectothermic animals, could serve as virus-amplifying hosts, while lessening the likelihood that numerous birds and non-primate mammals are competent reservoirs for CHIKV.	(Matusali et al., 2019)
100	Tonate virus	Togaviridae, Alphavirus	no	yes	yes		(Fischer et al., 2021)
101	Eestern equine encephalitis virus (EEEV)	Togaviridea, Alphavirus	no	no	no		(Sotomayor-Bonilla et al., 2017)

For each such virus, we examined the main papers describing the bat being the reservoir of this virus.

Another common type of research attempts to intentionally infect bats with human pathogens, such as Nipah virus (Middleton et al., 2007), various Coronaviruses (Watanabe et al., 2010; Munster et al., 2016), and Ebola (Zaire) virus (Swanepoel et al., 1996), among many others. Such research has revealed that the viruses can replicate and circulate in bats until they eventually die out. These experiments are insufficient to consider bats as reservoirs. If anything, they indicate that humans are reservoirs of potential bat-pathogens. Moreover, in some cases, such as Ebola, infection experiments have demonstrated that the virus can also infect other animals (e.g., mice) (Swanepoel et al., 1996). Intentional infection of the grey-headed fruit bat (*Pteropus poliocephalus*) with Nipah virus (Middleton et al., 2007) nicely demonstrated how bats contend with the virus up to full recovery (zero viruses isolated 21 days post-infection from urine or any other bat tissue), resulting in immunity (virus-neutralizing antibodies detected 15 days in all the tested bats). This response is probably owing to the bat’s unique immune system (discussed below).

To date, the evidence regarding the isolation of actual harmful pathogen viruses in bats is limited, with only a few well-known cases, including the Marburg virus that was isolated from *Rousettus aegyptiacus* fruit bats in Uganda (Towner et al., 2009), and Hendra virus (HeV) that was isolated from Australian fruit bats. In the case of the Marburg virus, some knowledge gaps regarding the full host range and circulation remain (Leendertz et al., 2016). In the case of Hendra, humans are infected by horses, which are supposedly infected by bats. Direct infection from bats is rare at most, as indicated by a serological survey of 128 people with prolonged and close contact with Pteropid bats, and in whom no evidence of infection with HeV (Selvey et al., 1996) was detected. This is important when considering the general public’s fear of bats (López-Baucells et al., 2018; MacFarlane and Rocha, 2020; Lu et al., 2021). Moreover, as pointed out by (Scott, 2001) virus isolation alone is not sufficient for considering an animal a reservoir, as evidence of transmission is also required. The mere detection of a virus in bats does not imply that spillover will occur, and many additional biological, ecological, and anthropogenic conditions must be in place for such an event to occur (Markotter et al., 2020). Some human pathogenic viruses are also known to infect and affect bats, including most lyssavirus species (Banyard et al., 2011), Tacaribe arenavirus (Cogswell-Hawkinson et al., 2012), and the Zwiesel bat banyangvirus (Kohl et al., 2020), among others that are known to harm bats.

As these examples show, bats are often perceived as reservoirs of viral diseases solely owing to being serologically positive, which merely means that the bats have survived the disease and developed an immune response to it (Yob et al., 2001; Li et al., 2005; Swanepoel et al., 2007). In other cases, a virus closely related to the human pathogen but not pathogenic to humans may be present in bats, which is not sufficient to make bats its reservoir. Whereas bats might have been the ancestral origin of such a human virus, an intermediate host in which the viral mutations occurred, and where the virus reached significant prevalence, is probably needed for zoonotic spillover of the virus to humans to occur. According to (Olival et al., 2017) not only bats but also primates and rodents have a higher proportion of observed zoonotic viruses compared to other groups of mammals. Species in other orders (e.g. Cingulata, Pilosa, Didelphimorphia, Eulipotyphla) also share a majority of their observed viruses with humans, but the data is limited in these less diverse and poorly studied orders.

### Unraveling the unique bat immune system

Interestingly, it seems that bats can contend with deadly viruses better than humans and most other mammals can. After over a century of focusing on the viruses that bats carry, there is increasing interest in understanding the uniquely potent bat immune system. Here, we summarize the findings to date, focusing on the ability of the bat immune system to fend off viral diseases.

Most early research focused on isolating the basic immune components of the innate and acquired bat immune system and comparing them with what was already known in mice and humans. Some of the main findings are as follows: Cells resembling follicular dendritic cells (FDCs) were described in *Pteropus giganteus* (Sarkar and Chakravarty, 1991) and macrophages, B cells, and T cells were identified in the spleen and lymph nodes of the Indian fruit bat. The complement cascade was found in *Tadarida brasiliensis* bats (Allen et al., 2009). A variety of immune cells, including lymphocytes, neutrophils, eosinophils, basophils, and macrophages, was also identified by morphological means in histological sections from the Brazilian free-tailed bat (*T. brasiliensis*) (Turmelle et al., 2010). The pattern recognition factor of toll-like receptors (TLR) was described in two species of fruit bat, *Pteropus alecto* and *R. leschenaultia* (Iha et al., 2010; Cowled et al., 2011), and found to be highly conserved between bats and other mammals. Several bat cytokine genes have now been characterized, including cDNAs corresponding to interleukin (IL)-2, IL-4, IL-6, IL-10, IL-12p40, and tumor necrosis factor (TNF) from *R. leschenaultii* (Iha et al., 2009), which both play an important role in the antiviral immune state. Bats have demonstrated a highly diverse antibody repertoire, exceeding that of most species and on a par only with humans and mice (Baker et al., 2010; Bratsch et al., 2011). Another study examined the interferon (IFN) signaling pathway following IFN production, to determine the importance of IFNs in inducing an “antiviral state” in bat cells through the simultaneous suppression of type I IFN and induction of type III IFN post virus infection (Virtue et al., 2011). The IFN systems in bats were later found to be highly diverse and much more complex than expected. A thorough review summarizing these innate and acquired immunological findings was published by (Baker et al., 2013b), showing that although bats appear to share most features of their immune system with other mammals, there are qualitative and quantitative differences in their immune responses.

Several of the early publications already provided initial evidence of one of the main characteristics of the bat immune system—a delayed immune response, on which we will elaborate later in discussion. McMurray and Thomas (McMurray and Thomas, 1979) and Paul (Paul and Chakravarty, 1986) found that T-cell proliferation as part of the immune response peaked at 120 h post-infection in comparison to 48 h in mice. Moreover, Chakraborty (Chakraborty, 1983) found that cell-mediated immunity in bats is slower than in other mammals. Prolonging the immune response was later found to be a beneficial antiviral strategy in bats (Hayman, 2019).

### Resistance versus tolerance in the bat immune response

A deeper understanding of the bat immune system was obtained using comparative genomics. Zhang et al. (Zhang et al., 2013) sequenced the genomes of two distantly related bat species (*P. alecto* and *Myotis davidii*) and revealed genetic evidence of a uniquely evolved immune system. Although some immune genes have been lost, others seemed to be under strong positive selection. Specifically, genes responsible for DNA damage checkpoints and repair pathways seemed to be undergoing accelerated positive selection. These authors hypothesized that flight-induced adaptations had inadvertently also affected the bat immune system. The strenuous and prolonged physiological efforts exerted during flight impose oxidative stress, resulting in severe DNA damage and the release of self-DNA fragments into the cytoplasm (Barzilai et al., 2002), somewhat similar to the DNA damage caused by a viral infection. Consequently, evolving an efficient DNA repair mechanism aimed at dealing with flight-induced cellular damage might have also enabled bats to fight off viral infections. Zhang et al. further hypothesized that these mechanisms may also be involved in the unique longevity of bats.

Additional bat genomes have as been studied, revealing new insights into the bat immune system (Zhang et al., 2013; Seim et al., 2013). Interestingly, one of the most important viral defense lines, namely the interferon (IFN) system, has been shown to vary greatly among bat species (Clayton and Munir, 2020). Interferons (IFNs) are secreted cytokines that induce an antiviral response by the host and are primarily responsible for inhibiting viral replication. Signaling pathways of IFN were already found in bats in 1969 (Stewart et al., 1969). New research has revealed a species-specific gene length size in bats, with much variability in functional responses, including permanent vs. stimulation-dependent secretion of IFNs, with different effects on the immune response: 1. Type I IFN locus has shortened in *Pteropus Alecto* (Zhou et al., 2016), but expanded in *Pteropus vampyrus* and *Myotis lucifugus* (Pavlovich et al., 2018); 2. Zhou et al., 2016) found a contraction of the type I IFN locus in the Australian black flying fox (*P. alecto*) and an unusual constitutive expression of IFN-α in these bats. Moreover, IFN type 3 in the same bat was induced in response to a viral infection; 3. Pavlovich et al., 2018) found a type I IFN complex in *Rousettus* bats, revealing an inhibitory signaling potential with no constitutive expression; 4. Banerjee et al., 2017) showed that while poly I:C treatment (imitating dsRNA stimulus which is usually associated with viral infection) induces the secretion of type I IFNs in both human and *Eptesicus fuscus* bat cells, the bat cells express much lower levels of these inflammatory mediators; and 5. Sarkis (Sarkis et al., 2018) found the induction of selective IFN stimulated genes in the common vampire bat (*Desmodus rotundus*). Some of these versatile responses led to the realization that the antiviral state achieved by a variety of IFN phenotypes in bats is also related to an anti-inflammatory response (see more in this recent review (Clayton and Munir, 2020).

The IFN system has also been shown to vary at the genetic regulation level. Xie and Li (Xie and Yang Li, 2020) demonstrated that a variety of bat species have a dampened interferon response owing to the replacement of the highly conserved serine residue in STING (stimulator of interferon genes), an essential adaptor protein in multiple DNA sensing pathways. This means that, in these species, the IFN response has substantially diminished, resulting in a reduced inflammatory response. Via the IFN antiviral cascade, the balanced reduction of inflammasome has started to be discovered.

### A restrained immune response serves better in contending with viruses

Recent findings suggest that a novel “trick” of the bat immune system might be that of the reduced inflammatory response that accompanies the antiviral response of the system. In recent years, evidence is accumulating that in addition to its antiviral abilities, the bat immune system is characterized by a general restrained response during inflammatory processes. One mechanism responsible for reducing the immune response is that of the complete and unique loss of the PYHIN gene that was found in *P. alecto* and *M. davidii* bats (Ahn et al., 2016). This family of proteins serves as important immune sensors of intracellular self and foreign DNA and as activators of the inflammasome and/or interferon pathways. This reduction aids in achieving a milder inflammatory response. Another example of a dampened pathway is related to the important inflammasome sensor NLR family pyrin domain-containing 3(NLRP3), which has been linked to both viral-induced and age-related inflammation. Ahn et al., 2019 found a dampened NLRP3-mediated inflammation in *P. alecto*,with implications for longevity and unique viral reservoir status. Recently, a diminished inflammatory signaling pathway was found in *P. alecto* and *M. davidii* bats (Goh et al., 2020).

As nicely summarized by Schneider et al. (Schneider and Ayres, 2008) there are two ways to survive infection: resistance and\or tolerance. It seems that bats have developed an excellent balance between the two: an enhanced host defense response, and immune tolerance through several different mechanisms (see (Irving et al., 2021) for a detailed review article). Suppressed inflammasome pathways—as noted above—contribute to immune tolerance in bats and a well-balanced reaction. In humans, the dysregulation of the immune system seems to be responsible for increasing the severity of illness in the acute phase of viral disease (Hope and Bradley, 2021). Bats, in contrast, contend better with deadly viruses and, despite a longer or slower time of reaction, they eventually overcome these viruses to reach full recovery and elimination of the pathogen. Recent studies have focused on bats’ ability to contend with some of the most notorious viruses, including Marburg virus (Guito et al., 2021), COVID-19 (Ruiz-Aravena et al., 2022), and others (Mandl et al., 2018). A restrained immune response has also been shown to be valuable regarding longevity (Kacprzyk et al., 2017; Gorbunova et al., 2020).

### Conclusions

When considering the interaction of bats with viruses, the time seems right for a paradigm shift. Many bats contend with a variety of deadly viruses better than other mammals. This ability has evolved over nearly 60 million years of adaptation to powered flight. Bats balance their immune response in such a way that it is slow but highly efficient, making them seropositive and immune to viruses. Following immunity, their chance of relapse, to the point of becoming contagious, is low. This is evident from the numerous studies cited above, which have not managed to isolate a viable virus from antibody-seropositive bat individuals; and it is also evident from intentional bat infections in which the virus was shown to disappear after up to one month. In most cases, bats thus carry and spread infectious agents during the limited time frame of their sickness before they overcome it. A spillover of viral pathogens can only occur when bats harbor the identical human pathogenic virus. However, many viruses carried by bats cannot infect humans without first undergoing a natural process of evolution, meaning that bats carry the ancestral viruses and not the human pathogen (Forni et al., 2017; Clayton and Munir, 2020; Latinne et al., 2020). This is also what is known so far for COVID-19 (Poon et al., 2004; Boni et al., 2020; Ruiz-Aravena et al., 2022; Frutos et al., 2022). We should seek to avoid the disruption of their natural habitats that are resulting from rapid urbanization, wildlife trade, and deforestation (Greger, 2007). This was neatly stated by Markotter et al. (2020), who wrote: “It is important to recognize the role of bats in zoonotic disease outbreaks and implement mitigation strategies to prevent exposure to infectious agents including working safely with bats. Equally important is the crucial role of bats in various ecosystem services. This necessitates a multidisciplinary One Health approach to close knowledge gaps and ensure the development of responsible mitigation strategies to not only minimize the risk of infection but also ensure the conservation of the species” (Markotter et al., 2020).

Bats' antiviral immunological abilities should be studied in greater depth, so that we, humans, may learn more about efficiently combating viral disease, aging, and cancer. The immense diversity of species in the Chiroptera makes the information gathered highly species-specific and therefore quite complex. Differences in the biology, ecology, and physiology of the different species constitute important factors that must be considered. Fortunately, such understanding is now growing in the scientific community (Foley et al., 2018; Mollentze and Streicker, 2020; Cockrell and An, 2021; Irving et al., 2021). Despite all of the above, bats are nonetheless frequently blamed for being virus reservoirs with the scientific literature driving the popular opinion. In light of the complex immunological and ecological phenomena that we have highlighted in this review, scientists should refrain from using generalizations such as: “… many of these terrible diseases are caused by viruses originated from bats” (Han et al., 2015); or headlines such as “Bats as reservoirs of severe emerging infectious diseases” or “Bats as vectors of diseases and parasites” (Klimpel and Mehlhorn, 2014). Like all animals, bats deserve a more accurate and scientific approach to the terminology applied to them (Puechmaille et al., 2021; Shapiro et al., 2021).
